# Impact of Backbone Fluorination on π-Conjugated Polymers in Organic Photovoltaic Devices: A Review

**DOI:** 10.3390/polym8010011

**Published:** 2016-01-12

**Authors:** Nicolas Leclerc, Patricia Chávez, Olzhas A. Ibraikulov, Thomas Heiser, Patrick Lévêque

**Affiliations:** 1 Institut de Chimie et Procédés pour l’Energie, l’Environnement et la Santé, ICPEES, Université de Strasbourg, CNRS, 25 rue Becquerel, 67087 Strasbourg, France; pchavez@unistra.fr; 2 Laboratoire ICube, DESSP, Université de Strasbourg, CNRS, 23 rue du Loess, Strasbourg 67037, France; ibraikulov@unistra.fr or ibraikulov@nu.edu.kz (O.A.I.); thomas.heiser@unistra.fr (T.H.); 3 National Laboratory Astana, Nazarbayev University, 53 Kabanbay Batyr Ave., Astana 010000, Kazakhstan

**Keywords:** fluorine, organic photovoltaics, conjugated polymer, optoelectronic

## Abstract

Solution-processed bulk heterojunction solar cells have experienced a remarkable acceleration in performances in the last two decades, reaching power conversion efficiencies above 10%. This impressive progress is the outcome of a simultaneous development of more advanced device architectures and of optimized semiconducting polymers. Several chemical approaches have been developed to fine-tune the optoelectronics and structural polymer parameters required to reach high efficiencies. Fluorination of the conjugated polymer backbone has appeared recently to be an especially promising approach for the development of efficient semiconducting polymers. As a matter of fact, most currently best-performing semiconducting polymers are using fluorine atoms in their conjugated backbone. In this review, we attempt to give an up-to-date overview of the latest results achieved on fluorinated polymers for solar cells and to highlight general polymer properties’ evolution trends related to the fluorination of their conjugated backbone.

## 1. Introduction

Solution-processed bulk heterojunction (BHJ) solar cells based on polymer composite active layers were first reported in 1995 [1]. After a slow initial increase in power conversion efficiencies (PCEs) [2,3], these devices have recently experienced a remarkable acceleration in performances, reaching PCE values above 10% [4]. This impressive progress is the outcome of the simultaneous development of more advanced device architectures and of optimized semiconducting polymers. As the physical mechanisms underlying the operation of a BHJ solar cell were progressively clarified, the requirements for a highly performing photoactive polymer were better defined. As a consequence, numerous polymers with continuously improved opto-electronic properties have been designed and have contributed significantly to this very positive evolution. The synthesis of pro-quinoïdal copolymers [5] or of copolymers alternating electron-donor (D) and electron acceptor (A) moieties [6] have, for instance, turned out to be particularly useful. Both chemical strategies allowed many research groups to fine-tune the frontier molecular orbital (FMO) energy levels by a suitable choice of the constitutive moieties. Other chemical and structural parameters have also been shown to strongly influence the polymer optoelectronic properties. Those parameters include the nature and position of the solubilizing alkyl side-chains, the backbone planarity (which can be enhanced by using ladder-type coplanar monomers) and the polymer molecular weight. Extensive descriptions on their impact on the solar cell performances can be found in several excellent review articles [7,8,9].

**Figure 1:**
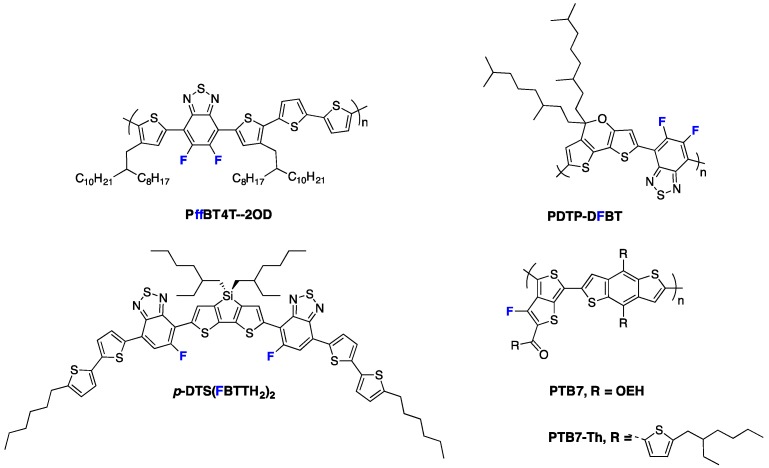
Chemical structures of highly efficient fluorinated materials for organic photovoltaics (OPV).

Fluorination of the conjugated polymer backbone has appeared more recently to be an especially promising approach for the development of efficient semiconducting polymers [10]. As a matter of fact, most currently best-performing semiconducting polymers are using fluorine atoms in their conjugated backbone. This is, for instance, the case for the **PffBT4T-2OD** polymer (see Figure 1) published recently by Yan and co-workers which holds the record PCE for single junction solar cells [4]. Another illustrative example is given by the **PDTP-DFTBT** copolymer (see Figure 1), which possesses a band-gap as low as 1.38 eV and attained an almost 8% PCE in single junction devices [11,12]. Also, the fluorinated derivatives of the **PTB7** polymer series (see Figure 1), which were designed following the pro-quinoïdal approach, achieved a PCE above 10% [13,14]. Jenekhe *et al.* demonstrated a PCE of 8% in all polymer solar cells using **PTB7** as electron donor material [15,16]. Last but not least, fluorination has also turned out to be efficient in designing highly performing molecular semiconductors. The fluorinated ***p*-DTS(FBTTH_2_)_2_** derivative, for instance (see Figure 1), is currently one of the best-performing small molecules for BHJ devices [17,18].

Possible physical mechanisms that may contribute to the remarkable increase in performances after backbone fluorination of different chemical systems have already been discussed in the literature. However, due to the large variety of molecular systems investigated so far and due to the complexity of the physics underlying the photovoltaic effect in organic BHJ devices, a general understanding of the impact of fluorination on the device performances is still lacking. This report therefore attempts to give an up-to-date overview of the latest results achieved on fluorinated polymers (mainly *p*-type) for solar cells and to highlight general trends observed in material properties that may be helpful for future polymer solar cell (PSC) developments. The following will cover, in particular, the impact of fluorination on (i) polymer synthesis; (ii) polymer frontier orbital energy levels; (iii) active layer morphology; (iv) charge transport properties; and (v) charge generation and recombination kinetics. A final paragraph is devoted to *n*-type fluorinated polymers (vi).

## 2. Synthesis of Fluorinated Conjugated Polymers

The synthesis of most fluorinated conjugated materials requires at least one additional chemical step compared to their non-fluorinated counter-derivatives. Indeed, the fluorine atoms need to be introduced before the functionalization step [10,19,20,21,22,23,24,25].

**Scheme 1:**
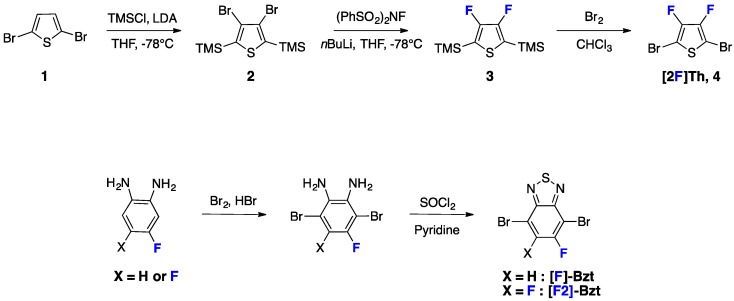
Synthetic procedures of some fluorinated units.

For instance, if we consider two of the most common building blocks in the conjugated polymer field, namely the thiophene electron-donating group [26,27,28,29,30,31,32] and the 2,1,3-benzothiadiazole [11,32,33,34,35,36,37,38,39,40,41,42] electron-accepting group, their fluorinated derivatives requires a minimum of three and one additional steps, respectively (see Scheme 1).

Interestingly, most of the fluorinated chemical moieties that are used as building blocks in conjugated polymers are now commercially available. However, due to their specific synthesis pathway, the production costs may exceed those of their non-fluorinated analogues up to 40 times.

**Scheme 2:**
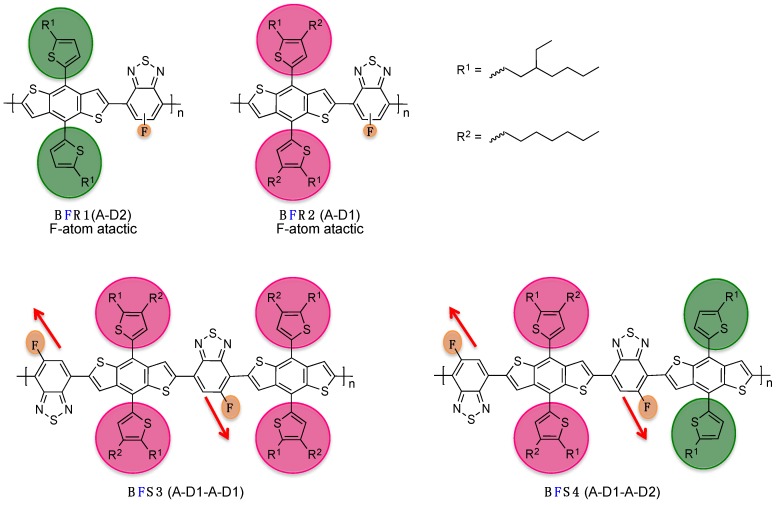
Chemical structures of regiorandom BFRx and regioregular BFSx (adapted with permission from [46]; Copyright 2014 American Chemical Society).

Another chemical issue related to the use of fluorinated derivatives occurs when considering asymmetric mono-fluorinated compounds: the regioregularity. Indeed, conventional polycondensation methods do not allow regioselectivity control when using asymmetric monomers [17]. Therefore, the lack of control of the monomer orientation along the conjugated backbone leads to regiorandom polymers [40,41,42,43,44] that are known to suffer from lower structural organization in solid state and consequently possess, most often, inferior charge transport properties [45]. An alternative stepwise chemical approach consists in synthesizing symmetric monomers from unsymmetrical fragments, thanks to their regiochemistry. This strategy allows the synthesis of polymers using, for instance, mono-fluorine aryl groups with well-controlled regioregularity and well-defined monomer alternation (see Scheme 2) [17,27,46].

Following this approach, Watkins *et al.* recently demonstrated the impact of the regioregularity control on the optoelectronic and structural properties in a series of D/A conjugated polymers (see Scheme 2) [46]. They showed, in particular, that the use of tailored A-D-A intermediate monomers enables control of the supramolecular interactions within regioregular **BFSx** polymer films. As a consequence, the photovoltaic performances, and related physical parameters, namely open-circuit voltage (*V*_oc_), short-circuit current density (*J*_sc_) and fill-factor (*FF*), were significantly improved in regards to regiorandom **BFRx** copolymers (see Entry 1, Table 1).

Regarding the chemical reactivity of fluorine species, if we consider that most of the conjugated polymers are synthesized by conventional palladium-catalyzed cross-coupling reactions such as Stille, Suzuki or direct heteroarylation reactions, the introduction of fluorine electron-withdrawing groups (EWG) on the chemical units should have, at minimum, no detrimental impact on the reaction yield and, at most, could increase the reaction rate. Indeed, in Suzuki and Stille polycondensation, the fluorine introduction in the halogen-aryl group is known to increase the reaction rate, as the EWG introduction into the halogenated aryl is known to increase the rate of the oxidative addition step. Since this oxidative addition is considered to be the rate-limiting step of the catalytic cycle, the EWG introduction in the halogenated aryl should increase the reaction yield [47].

**Table 1:** Characteristic properties of polymer solar cells.

Entry [reference]	Polymer/PCBM	*V*_oc_ (V)	*J*_sc_ (mA·cm^−2^)	*FF* (%)	PCE (%)
1 [46]	BFR2/PC_61_BM	0.89	7.25	60.6	3.91
	BFS3/PC_61_BM	0.90	10.8	61.0	5.67

**Figure 2:**
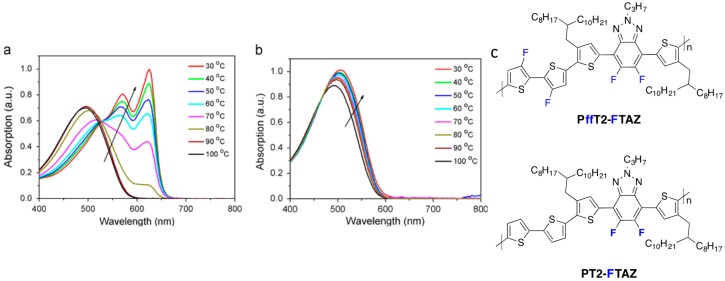
(**a**) UV–vis absorption spectra evolutions of PffT2-FTAZ in dichlorobenzene solution (cooling process, from 100 to 30 °C); (**b**) UV–vis absorption spectra evolutions of PT2-FTAZ in dichlorobenzene solution (cooling process, from 100 to 30 °C); (**c**) Chemical structures of the fluorinated copolymers (adapted with permission from [32]; Copyright 2015 Elsevier).

It is interesting to mention that, in contrast, when the fluorine substitution is made on the organometallic species, almost no impact on the reaction rate is observed. The opposite behavior is observed in direct heteroarylation polycondensation (DHAP) [48], in which the presence of EWG on the H-aryl group is known to increase the reaction rate [20,49]. However, despite this high cross-coupling polymerization reactivity, most of the literature on fluorinated polymers points to a polymer molecular weight decrease upon backbone fluorination [20,23,24,44]. This is clearly due to the higher planarity and non-covalent interactions (H^…^F, S^…^F and F^…^F) observed in fluorinated backbones. These factors are responsible for a significant solubility decrease of fluorinated polymers in regards to non-fluorinated analogues [5,36,38,41]. Indeed, more planar polymers tend to aggregate. Aggregation in solution is responsible for the strongly temperature-dependent absorption spectrum reported by some authors (Figure 2) [32]. The impact of fluorination on the morphology of pure polymer films and their blends with fullerenes will be discussed further below.

## 3. Influence of Fluorination on the Frontier Molecular Orbitals (FMO) of Conjugated Polymers: Experimental and Simulation Results

**Figure 3:**
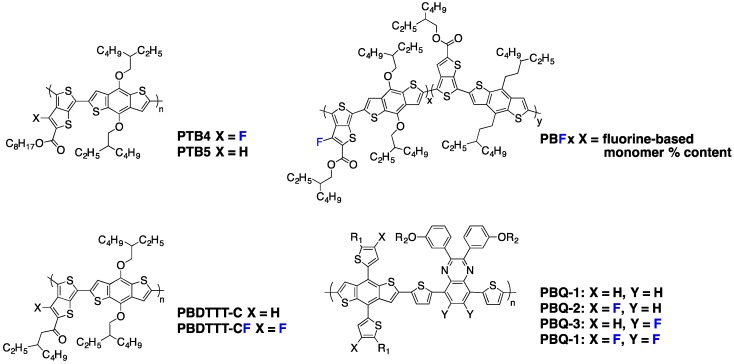
Chemical structures of some investigated polymers.

In the first publications on conjugated backbone fluorination in semi-conducting polymers for PSC, the introduction of fluorine atoms was mainly reported to decrease the HOMO (Highest Occupied Molecular Orbital) and LUMO (Lowest Unoccupied Molecular Orbital) energy levels. Indeed, fluorine is the chemical element with the highest electronegativity (EN = 4 in the Pauling scale). Its strong electron-withdrawing nature should lower both FMO energy levels as demonstrated theoretically on the cyano electron-withdrawing group by Heeger and Brédas and experimentally by Zyung and co-workers on a poly-*para*(phenylene-vinylene) (PPV) derivative [50,51]. Accordingly, in 2009, Yu and co-workers, following their **PTB** series investigations, described one of the first examples of efficient fluorinated polymers for PSC [10]. They showed that the introduction of a fluorine atom on the 3 position of the thieno[3,4-*b*]thiophene pro-quinoidal unit (see **PTBn** polymers in Figure 3) significantly lowers the HOMO and LUMO levels with only a minor impact on the band-gap, as reported in Entry 1, Table 2. Interestingly, as claimed by the authors, the replacement of a hydrogen atom by the small-sized fluorine atom (its Van der Waals radius, *r*_F_ = 1.35 Å, is only slightly larger than that of hydrogen, *r*_H_ = 1.2 Å) does not increase the steric hindrance along the conjugated backbone. The lowering of the HOMO level by 0.11 eV, going from **PTB5** to **PTB4** (Figure 3), leads to a 0.08 V increase in *V*_oc_, as summarized in Entry 1, Table 3. This result was expected since it has been established that the *V*_oc_ in donor/acceptor BHJ solar cells is proportional to the difference between the HOMO of the donor and the LUMO of the acceptor [52]. Changes in the other photovoltaic parameters (*J*_sc_, *FF*) induced by the backbone fluorination were attributed to differences in the active layer morphology, although the underlying physical mechanism remained unknown.

Broader evidence for the simultaneous lowering of both HOMO and LUMO levels upon fluorination was brought by Yu and co-workers during the same year [53]. They compared, among others, two **PTB** polymers still including a fluorine atom on the C3 position of the thieno[3,4-*b*]thiophene, but using side-chain carrying keto groups instead of the previously used ester groups (see **PBDTTT-C** and **PBDTTT-CF** in Figure 3). Both polymers possess identical band-gaps of 1.77 eV, but the FMO energy levels are downshifted by 0.1 eV for the fluorinated **PBDTTT-CF** polymer (see Entry 2, Table 2). The corresponding increase in *V*_oc_ contributes to the higher PCE of 7.73% observed for **PBDTTT-CF** (against 6.58% for **PBDTTT-C**) (see Entry 2, Table 3). Similar trends have been reported by other groups using various chemical compositions [54,55].

**Table 2:** FMO energy levels and band-gap values of some investigated polymers.

Entry [reference]	Polymer	HOMO (eV)	LUMO (eV)	*E*g_CV_ (eV)	*E*g_Opt_ (eV) ^a^
1 [10]	PTB4	−5.12	−3.31	1.81	1.63
	PTB5	−5.01	−3.24	1.77	1.62
2 [53]	PBDTTT-C	−5.12	−3.35	1.77	1.61
	PBDTTT-CF	−5.22	−3.45	1.77	1.61
3 [56]	PBF0	−5.22	-	-	1.57
	PBF100	−5.28	-	-	1.61
4 [23]	PBQ-1	−5.05	−3.29	1.76	1.64
	PBQ-2	−5.19	−3.43	1.73	1.66
	PBQ-4	−5.35	−3.55	1.80	1.73

^a^ From UV–visible absorption onset in solid state.

**Table 3:** Characteristic properties of polymer solar cells.

Entry [reference]	Polymer/PCBM	*V*_oc_ (V)	*J*_sc_ (mA·cm^−2^)	*FF* (%)	PCE (%)
1 [10]	PTB4/PC_61_BM	0.74	13.0	61.4	4.10
	PTB5/PC_61_BM	0.66	10.7	58.0	5.90
2 [53]	PBDTTT-C/PC_71_BM	0.70	14.7	64.1	6.58
	PBDTTT-CF/PC_71_BM	0.76	15.2	66.9	7.73
3 [56]	PBF0/PC_71_BM	0.72	16.7	56.0	6.69
	PBF100/PC_71_BM	0.79	16.7	63.0	8.36
4 [23]	PBQ-1/PC_71_BM	0.63	12.4	69.8	5.63
	PBQ-2/PC_71_BM	0.75	13.0	63.1	6.25
	PBQ-4/PC_71_BM	0.90	13.5	70.0	8.55

Following these studies, many publications reported a similar FMO energy level lowering effect through backbone fluorination. If the general trend of decreasing the HOMO level is respected, some differences appear in the LUMO level and in the energy band-gap evolutions. Recently, Cao and co-workers performed a systematic study of **PTB** copolymers with different amounts of fluorine decoration (called **PBFx**, see Figure 3) [56]. They synthesized five random copolymers by Stille polycondensation, adjusting the fluorine content by modulating the initial monomer feed ratio (*x* = 0, 25, 50, 75, 100). They deeply investigated the structure-property relationships in this series, from the physico-chemical properties to solar cell performances. As previously observed, the fluorine substitution slightly decreases the HOMO energy level of the resulting polymers, with a difference of 0.06 eV obtained between the most fluorinated (**PBF100**) and the non-fluorinated polymers (**PBF0**, Entry 3, Table 2). It is interesting to note that, unlike the previous reports by Yu *et al.* [53] discussed above, the optical band-gap is slightly increased upon backbone fluorination. A similar behavior has also been reported by other groups [27,57,58], demonstrating that the addition of a withdrawing fluorine atom may have more influence on the HOMO level than on the LUMO level. This result is somewhat surprising, as it is generally believed that the HOMO level of a D/A copolymer should be mostly controlled by the fluorine-free electron-donor moiety [6]. A tentative explanation for this counterintuitive experimental observation is given below. As shown in Entry 3, Table 3, the PCE increase upon backbone fluorination is not only due to the *V*_oc_ increase; however, its improvement from 0.72 to 0.79 V is again of significance in the PCE evolution [56].

Recently, Hou and co-workers reported a moderate band-gap copolymer in which they tuned the FMO energy levels by introducing fluorine atoms on different grafting sites (see **PBQ** series in Figure 3) [23]. A strong synergistic effect of the fluorination was observed by the authors. Indeed, the **PBQ-4** copolymer, including four fluorine atoms along the conjugated backbone, presents a far deeper HOMO level in regards to its non-fluorinated **PBQ-1** analogue and to the partially fluorinated **PBQ-2** and **PBQ-3** derivatives (Entry 4, Table 2). The 0.3 eV increase in ionization potential when going from **PBQ-1** to **PBQ-4** is in line with the 0.27 V *V*_oc_ increase measured on photovoltaic devices (Entry 4, Table 3). An auxochromic effect of the fluorine atom has also been observed by these authors, with a significantly enhanced extinction coefficient in solution and in solid state when the fluorine atom density along the conjugated backbone is increased. A similar effect has been previously observed by You and coworkers [59].

Several groups performed Density Functional Theory (DFT) calculations in order to get a deeper understanding of the experimental observations [23,25,27,28,31,38,39,40,57,60,61]. The necessarily limited number of monomers taken into account in the calculations does not, however, allow a quantitative comparison with the experiments. Also, neither the impact of side-chains nor the intermolecular interactions in thin films can be assessed, as the solubilizing side-chains are generally replaced by methyl groups to reduce the calculation time and are generally performed on single molecules.

**Figure 4:**
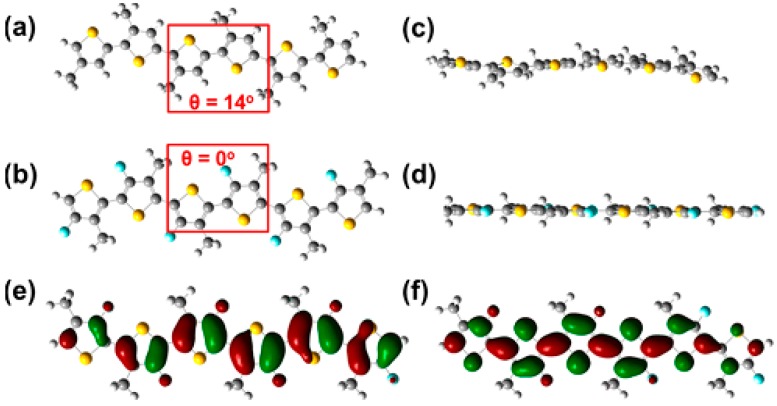
Top view of energy-minimized structures of a methyl-substituted hexameric (**a**) P3AT and (**b**) F-P3AT. Side view of energy-minimized structure of (**c**) a methyl-substituted P3AT and (**d**) FP3AT. HOMO (**e**) and LUMO (**f**) distributions for the energy minimized structure of F-P3AT. All calculated using DFT at the B3LYP/6.31G (d,p) level (adapted with permission from [27]; Copyright 2015 American Chemical Society).

Nevertheless, the experimentally observed decrease of both HOMO and LUMO levels is well reproduced by the DFT calculations. Moreover, the surface contour plots of the HOMO and LUMO levels are predicted to be rather unaffected by the fluorination [23], although the LUMO is sometimes found to be slightly more delocalized [25]. DFT calculations also reveal a higher planarity of the backbone of fluorinated polymers (as, for instance, in Figure 4).

The origin of the enhanced planarization induced by the fluorine atoms is still under debate. In-depth quantum chemical calculations highlight the hydrogen-fluorine interactions as the main reason to lock the conformation of the polymer backbone into a more planar state [62]. Other reports claim that the S^…^F and F^…^F interactions may influence the planarization [27,63,64].

**Figure 5:**
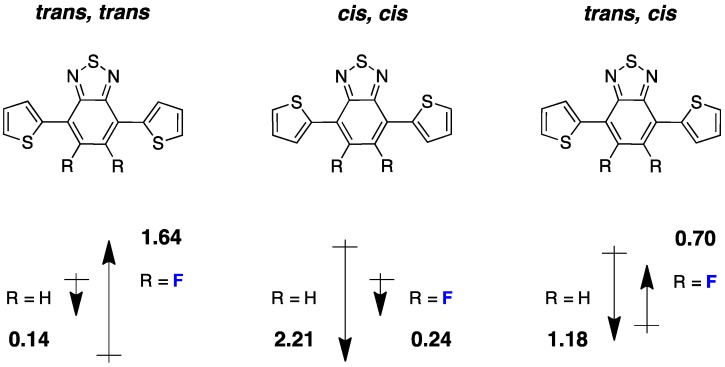
Chemical structure of DTBT (R = H) and DTF2BT (R = F) and the calculated dipole moment for each configuration (adapted with permission from [38]; Copyright 2015 American Chemical Society).

Recently, DFT calculations have been combined with other methods (time-dependent DFT and/or semi-empirical models to access the excited states) to estimate the dipole moment of fluorinated segments and evaluate its possible influence on the photovoltaic performances. A recent example of such an approach was made for the D/A copolymers based on 4,7-dithieno-2,1,3 benzothiadiazole (**DTBT**) moieties [38,39]. In a study of single crystals of **DTBT** trimers, McCulloch *et al.* compared non-fluorinated and fluorinated (**DTF2BT**) trimers (Figure 5) and showed that the calculated dipole moment correlates with the crystal structure [38].

**Figure 6:**
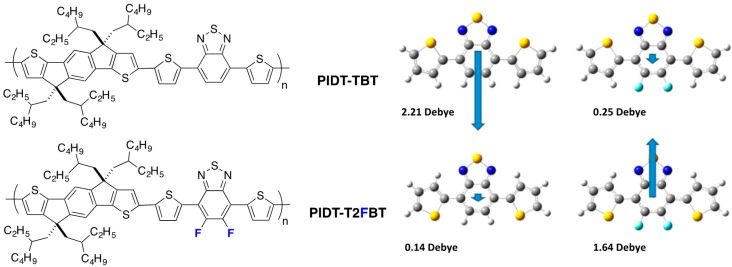
Chemical structure of PIDT-TBT and PIDT-T2FBT and the calculated dipole moment for the DTBT and DTF2BT trimers (adapted with permission from [39]; Copyright 2013 American Chemical Society).

In single crystals, the *trans, cis* configuration is the most prominent one and shows a dipole moment significantly larger for the non-fluorinated trimer. An in-depth structural study links this larger dipole moment with the observed crystal structure and to the distribution of conformational orientations in solutions with solvents of different polarity. The previously described trimers were used as building blocks in copolymers together with indacenodithiophene (**IDT**) (Figure 6).

In the case of the non-fluorinated **DTBT**, the *cis, cis* conformation is less planar and presents a large dipole moment while the *trans, trans* conformation has a negligible one (Figure 6). The authors state that when polar solvents are used to deposit films (for transistors or photovoltaic cells elaboration), the stabilization of the non-planar *cis, cis* conformation may dominate. The trend is opposite for **DTF2BT** and the planar *trans, trans* configuration could be favored for this material in thin films elaborated from polar solvents. The authors extrapolate this conclusion to copolymers **PIDT-TBT** and **PIDT-T2FBT** and anticipate less nonplanar defects in the case of the fluorinated copolymer.

Interestingly, in the same study, the authors point out the counterintuitive trend observed in the frontier energy levels in fluorinated D/A copolymers. As in alternating D/A copolymers, the HOMO is, in general, associated with the electron-rich fragment of the molecule (here the **IDT**) and the LUMO with the electron-poor benzothiadiazole fragment of the molecule (**BT**), and the authors highlight that one may naively think that the withdrawing effect of the fluorine may affect the LUMO more than the HOMO. They show with a close look at the calculated HOMO and LUMO surfaces of **PIDT-T2FBT** that fluorine can participate in the mesomeric donation of electronic density from its lone pairs.

In order to clarify the HOMO energy level decrease upon backbone fluorination, we also performed DFT calculations on simple trimers of benzothiadiazole sandwiched by two thiophene (**T**) units (as in reference [38]), the central benzothiadiazole unit bearing either none, one or two fluorine atoms. We applied the same method as in reference [38] (DFT at the B3LYP/6-31G* level of theory in vacuum) and found, like McCulloch *et al.*, that the preferential calculated conformation for the three types of trimers is *trans, trans*. The calculated frontier energy levels for the three trimers can be found in Figure 8.

**Figure 7:**
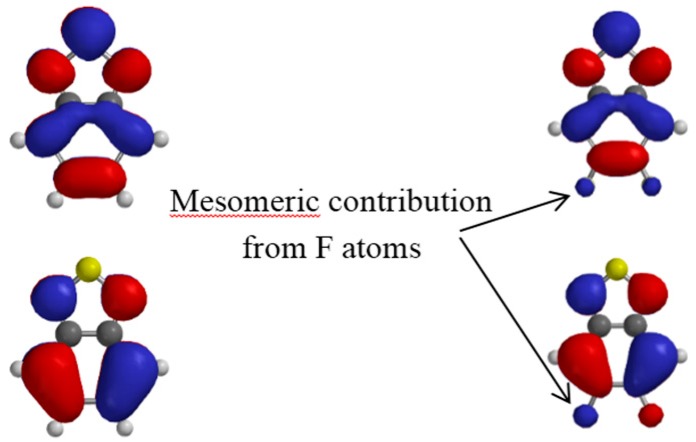
Calculated LUMO (**top**) and HOMO (**bottom**) isovalue contour plots for BT (**left**) and 2FBT (**right**). The arrows indicate the mesomeric contribution of the F atoms in 2FBT.

The mesomeric contribution from the F atoms can be seen in Figure 7 where we plot the HOMO and LUMO surface contours for the benzothiadiazole unit bearing no fluorine atom (**BT**) and two fluorine atoms (**2FBT**).

**Figure 8:**
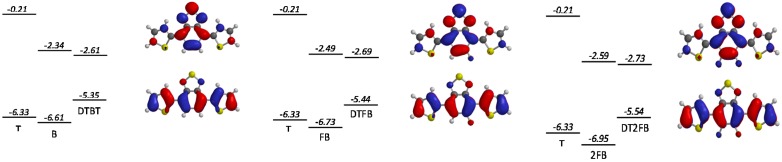
Calculated LUMO and HOMO levels (relative to the vacuum energy level) for the different moieties and the trimers together with the LUMO (**top**) and HOMO (**bottom**) isovalue contour plots. From the left to the right: DTBT, DTFBT and DT2FBT.

Interestingly, the introduction of fluorine atoms on the benzothiadiazole moiety lowers both the HOMO and LUMO energy levels. This effect is most pronounced for **2FBT** and can be associated with changes in the degree of hybridization between the FMO of **T** and **BT** upon fluorination. In general, the magnitude of hybridization depends on the energy difference of the involved molecular orbitals as well as on the orbital overlap. As the investigated trimers are all planar (dihedral angles lower than 5°), the latter parameter should not be significantly affected by the fluorination. On the other hand, the energy difference between the LUMO levels of **T** and of the central unit is large (more than 2 eV), hindering hybridization, while the corresponding HOMO level energy difference is significantly lower (less than 0.62 eV), enhancing hybridization. As a consequence, the LUMO of the trimer is close to the deepest LUMO of the constituting moieties (the LUMO of the central unit). As the latter is lowered upon fluorination, the same holds for the fluorinated trimers. On the opposite side, the HOMO level hybridization is more pronounced, making the trimer HOMO lie above the **T** HOMO. However, as fluorination deepens the benzothiadiazole central moiety HOMO level, the hybridization magnitude is reduced in **FBT** and **2FBT**. Consequently, the HOMO of the fluorinated trimer lies closer to the **T** HOMO level. These calculations are reproduced in Figure 8 (the scale is respected) together with the HOMO and LUMO surface contour plots of the trimers. Although this description above does not allow a quantitative comparison with the experimentally observed energy changes upon fluorination, it brings some insight into the mechanism that underlies the effect of fluorination on both frontier orbital energy levels.

Other chemical strategies have been reported to have a similar impact on the FMO energy levels of conjugated materials [22,65,66]. However, fluorination of the conjugated backbone appears to be most effective in producing high efficiency photovoltaic performances [31,33,67,68]. Actually, as shown in Table 3, *V*_oc_ is not the only parameter to be affected by fluorination. The *J*_sc_ and *FF* are also significantly modified. As the latter parameters are sensitive to the active layer morphology, the charge carrier transport properties, and the charge carrier generation and recombination rates, the impact of fluorination on these properties will be described in more detail below.

## 4. Impact of Fluorination on the Active Layer Morphology

The active layer morphology in bulk heterojunction solar cells is well known to be crucially important for the device performances as it determines, to a large extent, the free charge carrier generation rate as well as charge transport and collection efficiencies. Critical morphological parameters are essentially the average donor and acceptor domain size, the domain purity and microstructure, as well as the D/A interface properties. A number of recent reports have shown that fluorination of conjugated polymer backbones can have a strong impact on one or several of these properties, often leading to a more favorable morphology and to improved photovoltaic performances. Adding fluorine heteroatoms has, for instance, been found to enhance the domain purity (lowering the charge carrier recombination rate) [25,59,67], reduce the domain size [28,32,59,67], enhance the structural order [24,25,28,29,59,61,69,70,71] and promote polymer face-on orientation at the interface with the bottom electrode [28,32,59,61,67,69,72] (increasing the out-of-plane hole mobility) or induce a face-on orientation at the D/A interface [69,73]. However, these effects can be more or less pronounced or even reversed [27,33,67], depending on the molecular structure of the conjugated building blocks as well as on the nature and positioning of the solubilizing side-chains. Predictive modeling of the impact of fluorination on the morphology remains, thus, beyond reach. Also, the driving forces that often allow fluorination to trigger advantageous morphological changes (for photovoltaic devices) are still poorly understood and merit further theoretical and experimental investigations [73]. This paragraph tries, nevertheless, to give a representative overview of recent results on the impact of fluorination on the active layer morphology.

**Figure 9:**
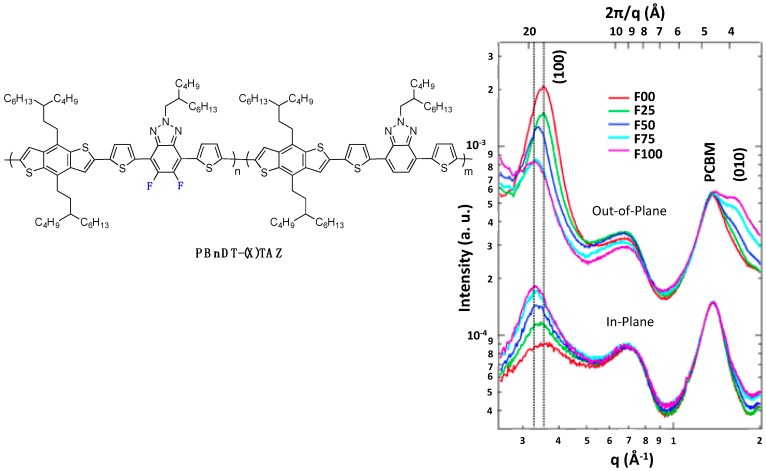
(**left**) Chemical structure of PBnDT-(X)TAZ polymers, (**right**) GIWAXS data of polymer/PCBM blend films. The (100) lamellar, PCBM, and (010) π-π stacking peaks are labeled. In-plane and out-of-plane data set offset for clarity (adapted with permission from [69]; Copyright 2014 American Chemical Society).

As already mentioned above, one frequently observed effect of fluorination is a higher planarity of the conjugated backbone [27,32,61,70]. As may be expected, this property has often (although not systematically [73]) been found to induce stronger π-π stacking and higher crystallinity of the fluorinated polymers in the solid phase [25,61,69,70]. You *et al.* observed, for instance, a systematic increase in π-π stacking with increasing fluorine content in a series of **PBnDT-(X)TAZ** polymers (see Figure 9) [69].

**Figure 10:**
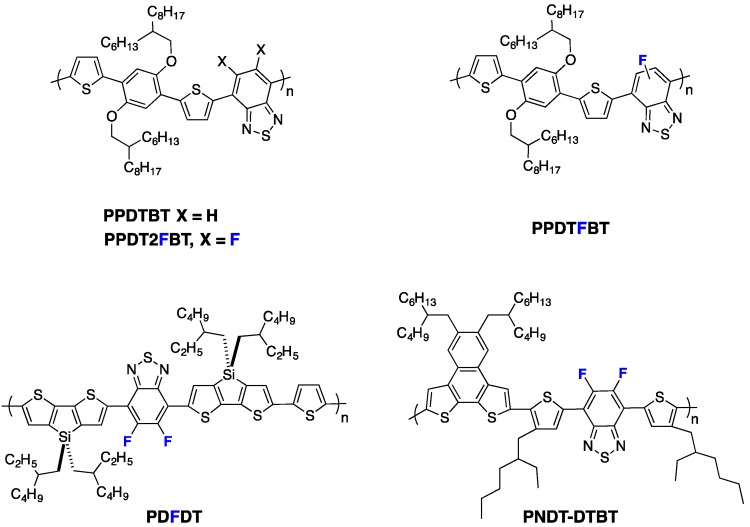
Chemical structures of some fluorinated polymers.

As a result, the photovoltaic device performances increased with the fluorine content (see Entry 1, Table 4). The higher out-of-plane hole mobility was shown to contribute most to these improved photovoltaic performances. Similarly, Woo *et al.* [70] reported reduced π-π stacking distances upon fluorination of **PPDTBT** (see Figure 10), with a pronounced impact on the charge transport in thick active layers (up to 1 µm thick) and thus on the photovoltaic device performances (see Entry 2, Table 4). Moreover, in their investigation, a computational study led them to attribute both the higher backbone planarity and the more intense interchain packing to F^...^H and F^...^S interactions. A related observation on **PDFDT** (see Figure 10) polymers was reported by Noh *et al.* [61]. Opposite examples exist, however, as well. Abe *et al.*, for instance, reported that π-π stacking in **PNDT-DTBT**-based blends (see Figure 10) remained weak, independent of fluorine substitution [73].

**Table 4:** Characteristic properties of polymer solar cells and charge carrier mobility measured on single carrier Space Charge Limited Current (SCLC) devices.

Entry [reference]	Polymer/PCBM	µ_h_ (cm^2^·V^−1^·s^−1^)	*V*_oc_ (V)	*J*_sc_ (mA·cm^−2^)	*FF* (%)	PCE (%)
1 [69]	PBnDT-00TAZ/PC_61_BM	1.7 × 10^−4^	0.73	11.3	46,6	3.84
	PBnDT-25TAZ/PC_61_BM	2.8 × 10^−4^	0.74	12.3	54.3	4.94
	PBnDT-50TAZ/PC_61_BM	5.5 × 10^−4^	0.76	12.4	62.3	5.92
	PBnDT-75TAZ/PC_61_BM	8.0 × 10^−4^	0.78	12.2	64.9	6.18
	PBnDT-100TAZ/PC_61_BM	1.2 × 10^−3^	0.80	12.7	70.6	7.17
2 [70]	PPDTBT/PC_71_BM	3.2 × 10^−4^	0.70	11.7	63.0	5.17
	PPDTFBT/PC_71_BM	5.5 × 10^−4^	0.73	13.3	69.0	6.64
	PPDT2FBT/PC_71_BM	3.0 × 10^−3^	0.79	16.3	73.0	9.39

The stronger π-π stacking interactions can counterweigh the steric hindrance of alkyl chains, allowing the introduction of relatively long and branched chains (up to C_24_H_49_) without jeopardizing the formation of crystalline lamellae [59]. Concomitantly, in some cases the inter-lamellar spacing has been observed to be enlarged as a consequence of more extended side-chain conformation. For the **PBnDT-(X)TAZ** polymer series investigated by You *et al.* [69], stronger π-π stacking was accompanied by an increase in the inter-lamellar spacing (left-shift of (100) Grazing-Incidence Wide-Angle X-ray Scattering (GIWAXS) peak position with increasing fluorine content, Figure 9), giving evidence for side-chain reorientation.

**Figure 11:**
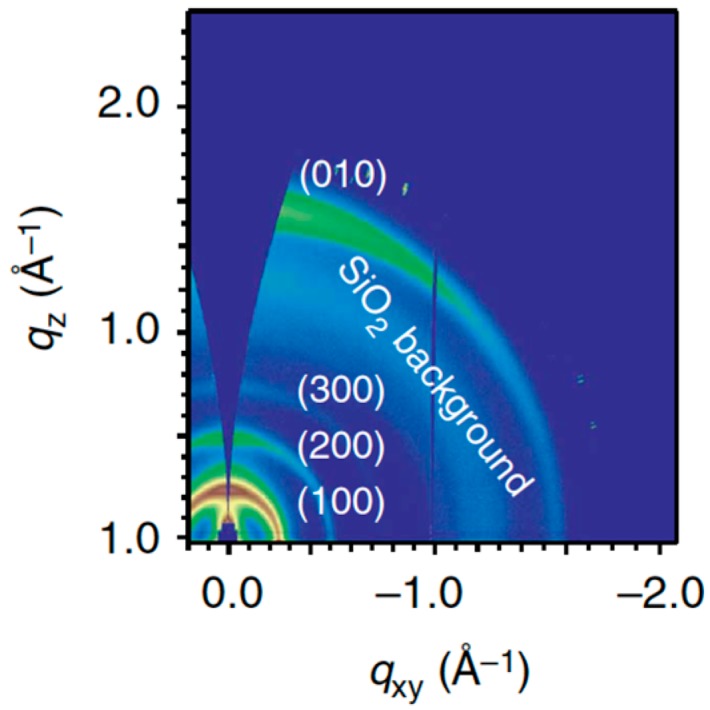
Two-dimensional (2D) GIWAXS pattern of a pure PffBT4T-2OD film (adapted with permission from [4]; Copyright 2014 Nature Publishing Group).

The orientation of the crystalline domains with respect to the bottom electrode is another critical parameter for solar cells. Indeed, charge transport in semi-crystalline polymers can be strongly anisotropic. In the case of a lamellar microstructure, the local mobility anisotropy (ratio between the mobility respectively along or perpendicular to the lamellae) may differ by orders of magnitude [74]. A dominantly parallel orientation of the lamellae along the substrate therefore hinders out-of-plane charge transport and leads to poor charge collection [75]. One of the most striking and, presumably, the most effectual consequence of fluorination appears to be the frequently observed presence of perpendicularly oriented lamellae [28,32,69,70,72]. The corresponding out-of-plane orientation of the π-π stacking direction leads to a high out-of-plane hole mobility, which opens the route towards thicker active layers without loss in fill factor. This is the case, in particular, for the **PffBT4T-2OD** polymer (see Figure 1) reported by Yan *et al.* [4], and which currently holds the record power conversion efficiency. As illustrated in Figure 11, GIWAXS investigations revealed the coexistence of out-of-plane- and in-plane-oriented lamellae. The latter give rise to the favorable out-of-plane π-π stacking responsible for good charge collection efficiency. The relative amount of in-plane *versus* out-of-plane lamellae appears to be strongly dependent on the film processing conditions. It is likely that small-sized crystalline aggregates preformed in solution adopt randomly one of both orientations during film-casting. A comparable behavior has been observed by You *et al.* [69] on the **PBnDT-(X)TAZ** polymer series (see Figure 9).

**Figure 12:**
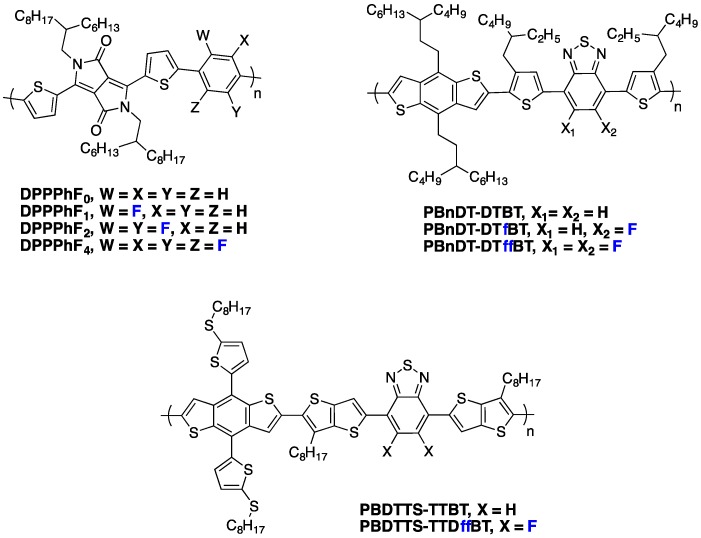
Chemical structures of some fluorinated polymers.

In some cases, however, fluorination has led to the opposite result, *i.e.*, a more pronounced parallel orientation of the lamellae, leading to significantly higher in-plane mobilities [24,29,33]. For instance, by investigating the influence of fluorination in **PDPP[T]_2_-TPT** polymers (see Figure 12), Thelakkat *et al.* observed stronger π-π stacking and larger inter-lamella distances, similar to what has been reported in other fluorinated polymers. In their case, however, no GIWAXS signature for out-of-plane-oriented π-π stacks could be observed. This result is s more surprising as a pronounced aggregation in solution for the fluorinated derivatives was also seen, suggesting that different film processing conditions may be at the origin of these conflicting observations.

The investigation by Abe *et al.* [74] of the molecular orientation of three different electron-donating polymers (with or without fluorine-substituent atoms) using resonant soft X-ray scattering did not corroborate the link between backbone fluorination-induced preferential face-on orientation at the polymer/electrode interface and enhanced device performances. Rather, a preferential polymer face-on orientation at the interface with the fullerene phase was observed. The latter was found to correlate well with the device performances, possibly due to more favorable charge generation and recombination kinetics at the D/A interface [76].

Polymer domain purity and average size are additional important parameters for the device performances, considering that high phase purity facilitates the charge separation and transport more effectively. Both parameters have been found to be altered by fluorination. A higher purity upon fluorination has, for instance, been reported by You *et al.* [59] for **PBnDT-DTffBT** (see Figure 12). A similar result was obtained by Jo *et al.* [67] on **PBDTTS-TTffBT** (see Figure 12), a medium band-gap polymer. A reduced solubility of PCBM in the polymer was suggested to be at the origin of this behavior [59]. You and co-workers found, in particular, that the miscibility of PCBM derivative in polymer is highest for non-fluorinated **PBnDT-DTBT** polymer at approximately 21% by weight and reduces to 16% and 12% for mono- and di-fluorinated copolymers **PBnDT-DTfBT** and **PBnDT-DTffBT**, respectively. The authors partly correlate this polymer domain purity evolution with the reduced para-crystallinity in fluorinated polymers.

On the other hand, similar investigations performed on **PBnDT-FTAZ**-based (see Figure 9) copolymers did not confirm this observation. Rather, the improved device performances upon fluorination were seen to correlate with changes in the molecular ordering within the domains. Thus, although fluorination has been frequently seen to induce advantageous morphological changes to the active layer, the complex molecular interactions involving both the solubilizing side-chains and conjugated backbones do not allow us to draw a general rule for the behavior of fluorinated polymers/fullerene blends.

## 5. Charge Transport Properties in Fluorinated Polymers

Charge transport in semi-conducting polymers remains an important bottleneck towards high efficiency PSCs. The influence of backbone fluorination on charge transport is strongly correlated to changes in the thin film morphology (see above) and has been investigated by a number of research groups. In particular, Noh *et al.* studied the field-effect hole mobility in **PDFDT** (see Figure 10), a polymer based on dithienosilole and difluorobenzothiadiazole moieties [61]. The authors achieved a hole mobility as high as 2.6 cm^2^·V^−1^·s^−1^ after optimization, using poly(methyl methacrylate) (PMMA) as a gate dielectric in top gate-bottom contact devices. The polymer exhibits an even more remarkable hole mobility of 9.0 cm^2^·V^−1^·s^−1^ when using a high-*k* P(VDF-TrFE) ferroelectric polymer as a gate dielectric. These mobility values are significantly larger than those reported previously for its non-fluorinated equivalent (3 × 10^−3^ cm^2^·V^−1^·s^−1^) [77]. Such a pronounced effect of fluorination may be attributed to the higher backbone planarity, which allows stronger π-stacking in solid state.

**Figure 13:**
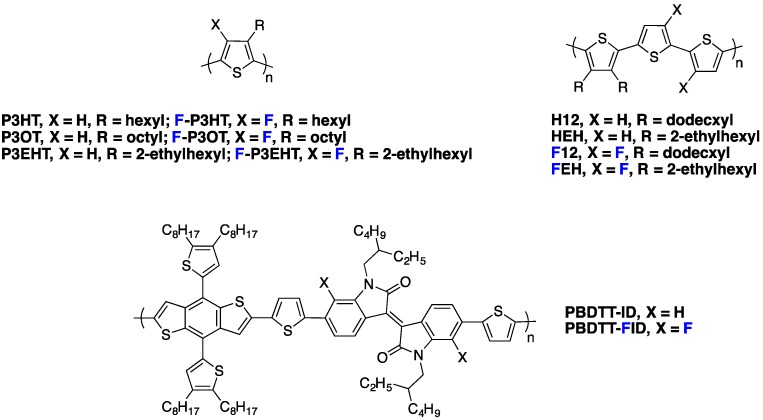
Chemical structures of some fluorinated polymers.

Recently, Heeney *et al.* [27] investigated the fluorination impact on charge carrier mobility in polyalkylthiophene derivatives (see Figure 13). They obtained an average field-effect hole mobility as high as 0.7 cm^2^·V^−1^·s^−1^ for the fluorinated **F-P3OT** polymer, which they attributed as well to a more planar fluorinated polymer backbone arrangement, corroborating previous reports [78]. Even if these polymers have not been used in PSC, the results highlight that fluorinated conjugated polymers are good candidates for hole transporting materials.

As photovoltaic devices require high charge carrier mobilities in the out-of-plane direction, SCLC (Space Charge Limited Current) devices have been frequently used to probe the charge carrier mobility in photovoltaic materials. Peng *et al.* [77] reported a SCLC study on two equivalent isoindigo polymers, the **PBDTT-ID** and the fluorinated **PBDTT-FID** derivative (see Figure 13). Fluorination resulted in a vertical mobility enhancement by a factor of five (from 6.5 × 10^−4^ to 3.2 × 10^−3^ cm^2^·V^−1^·s^−1^) and in a significant increase in *J*_sc_ and *V*_oc_ (see Entry 1, Table 5). Correspondingly, the PCE increased from 4.76% to 5.52% upon fluorination and reached 7.04% after additional device optimization (inverted structure).

**Table 5:** Characteristic properties of polymer solar cells and charge carrier mobility.

Entry [reference]	Polymer/PCBM	µ_h_^a^ (cm^2^·V^−1^·s^−1^)	*V*_oc_ (V)	*J*_sc_ (mA·cm^−2^)	*FF* (%)	PCE (%)
1 [79]	PBDTT-ID/PC_71_BM	6.5 × 10^−4^	0.88	8.95	60.0	4.76
	PBDTT-FID/PC_71_BM	3.2 × 10^−3^	0.94	9.62	61.0	5.52
2 [28]	HEH/PC_71_BM	2.2 × 10^−5^	0.80	4.04	43.0	1.39
	FEH/PC_71_BM	3.0 × 10^−4^	0.87	9.82	61.0	5.20
3 [32]	PT2-FTAZ/PC_71_BM	3.0 × 10^−4^	0.73	5.3	55.0	2.8
	PffT2-FTAZ/PC_71_BM	1.7 × 10^−3^	0.80	13.3	69.0	7.8
4 [80]	2F/PC_71_BM	4.4 × 10^−4^	0.80	14.1	63.0	7.11
	3F/PC_71_BM	5.7 × 10^−4^	0.82	15.7	71.0	9.14
	4F/PC_71_BM	2.7 × 10^−4^	0.82	13.3	59.0	6.43
5 [39]	PIDT-TBT/PC_71_BM	1.3 × 10^−1^ ^b^	0.79	9.4	50.0	3.7
	PIDT-T2FBT/PC_71_BM	6.0 × 10^−2^ ^b^	0.86	8.8	59.0	4.4

^a^ mobilities measured by the SCLC method; ^b^ mobilities measured by FET in the linear regime.

Similar results were obtained by Jo *et al.* on fluorinated polythiophene derivatives (see Figure 13) [28], exhibiting more pronounced backbone planarity and shorter interchain distances (measured by GIWAXS on thin films) than their non-fluorinated counterparts. Moreover, for the **FEH** derivative, a major fraction of the polymer adopted a face-on orientation in blends with PC_71_BM, contributing to effective charge transport in the out-of-plane direction. As a result, the SCLC hole mobilities in fluorinated polymer/PC_71_BM blends were significantly higher than in non-fluorinated polymer/PC_71_BM blends (3.0 × 10^−4^ and 2.2 × 10^−5^ cm^2^·V^−1^·s^−1^, respectively). Concurrently, the PCE increased from 1.4% to 5.2% for the fluorinated derivative, with *J*_sc_ and *FF* increasing from 4.0 mA·cm^−2^ and 43% to 9.8 mA·cm^−2^ and 61%, respectively (see Entry 2, Table 5).

**Figure 14:**
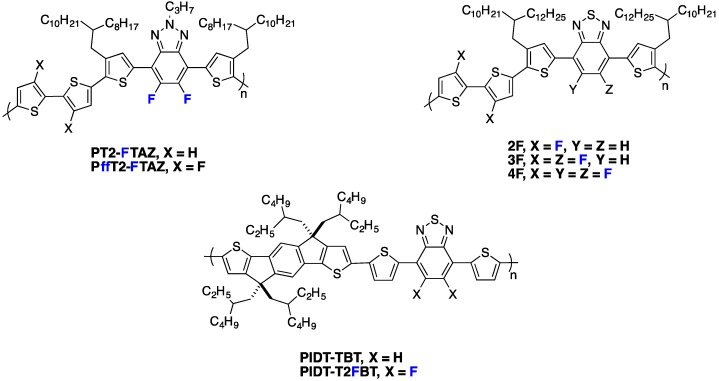
Chemical structures of some fluorinated polymers.

In a different work, Woo *et al.* investigated the fluorination of benzothiadiazole in a D/A copolymer including thiophene-dialkoxybenzene-thiophene as an electron-donor comonomer [70,81]. The authors could demonstrate, through the investigation of three different polymers (non-fluorinated **PPDTBT**, mono-**PPDTFBT** and di-fluorinated **PPDT2FBT** copolymers, see Figure 10), that the SCLC mobility increased with the fluorine substitution for both electrons and holes. The hole mobility increased by one order of magnitude (from 3.2 × 10^−4^ to 3.0 × 10^−3^ cm^2^·V^−1^·s^−1^) while the electron mobility increased from 2.8 × 10^−4^ to 1.5 × 10^−3^ cm^2^·V^−1^·s^−1^ upon di-fluorination. They could thereby achieve a balanced charge transport in blends with PC_71_BM. The resulting PCE exceeded 9% in single-cell devices with a 300 nm active layer thickness (see Entry 2, Table 4). The high *FF* measured for such a thick active layer (above 70%) is in line with the high and balanced vertical charge carrier mobilities. Enabling high PCEs with such thick active layers is of considerable interest for industrial applications as it should facilitate the production of organic photovoltaic (OPV) modules by roll-to-roll processing. This feature is well illustrated by the recent work of Yan and co-workers [32]. The authors compared di-fluorinated **PT2-FTAZ** and tetra-fluorinated **PffT2-FTAZ** (see Figure 14) and showed the SCLC hole mobility in **PffT2-TAZ**/PC_71_BM blends (1.7 × 10^−3^ cm^2^·V^−1^·s^−1^) to be significantly higher than that of **PT2-TAZ**/PC_71_BM blends (3.0 × 10^−4^ cm^2^·V^−1^·s^−1^). Again, this advantageous property allowed them to fabricate high efficiency solar cells (PCE = 7.8%) with thick active layers (≈250 nm) without sacrificing the *FF* (≈70%) (see Entry 3, Table 5).

For the same polymer family, Jo *et al.* investigated the influence of fluorination of both electron-donor and electron-acceptor moieties [80] including either two, three or four fluorine atoms along the conjugated monomer backbones (see **2F**, **3F** and **4F** copolymers in Figure 14). The field-effect hole mobility in pure polymer films was found to increase with the number of fluorine atom substitutions (up to 0.62 cm^2^·V^−1^·s^−1^ for polymer substituted with four fluorine atoms). On the other hand, the SCLC hole mobility in blends with PC_71_BM was higher for the polymer with three fluorine atoms along the backbone. Consistently, the solar cells performed best using **3F** polymer (PCE = 9.14%), exhibiting a higher *J*_sc_ and *FF* with respect to **2F** and **4F** (see Entry 4, Table 5). Other recent reports confirmed the good photovoltaic performances obtained with thick active layers using fluorinated polymers [4,33,82].

Nevertheless, a few opposite results, with fluorination having a detrimental impact on charge transport, have been reported as well. For instance, a significantly lower field-effect hole mobility (in pure polymers) upon fluorination was reported by McCulloch *et al.* in a series of alternating fluorinated and non-fluorinated polymers (see **PIDT-TBT** and **PIDT-T2FBT** in Figure 14) [39]. The reduced mobilities were ascribed by the authors to an increased surface roughness for the fluorinated polymer films, as observed by atomic force microscopy (AFM). Interestingly, despite the lower in-plane mobility, the performances of solar cells were better with fluorinated polymer, mainly due to a significant increase in *V*_oc_ and *FF* (see Entry 5, Table 5). If the positive change in *V*_oc_ at that time was expected, the origin for the *FF* improvement was not clarified by the authors. Unfortunately, SCLC measurements, which would have been necessary to verify whether fluorination yields a higher out-of-plane mobility and therefore a better *FF*, were not included in the report. We note that other examples of a simultaneous lowering of the field-effect hole mobility with an increase in *FF* and *PCE* upon fluorination have been reported in the literature and may similarly be signatures for a morphology that favors out-of-plane charge transport [83].

## 6. Charge Carrier Generation and Recombination in Fluorinated Polymers

**Figure 15:**
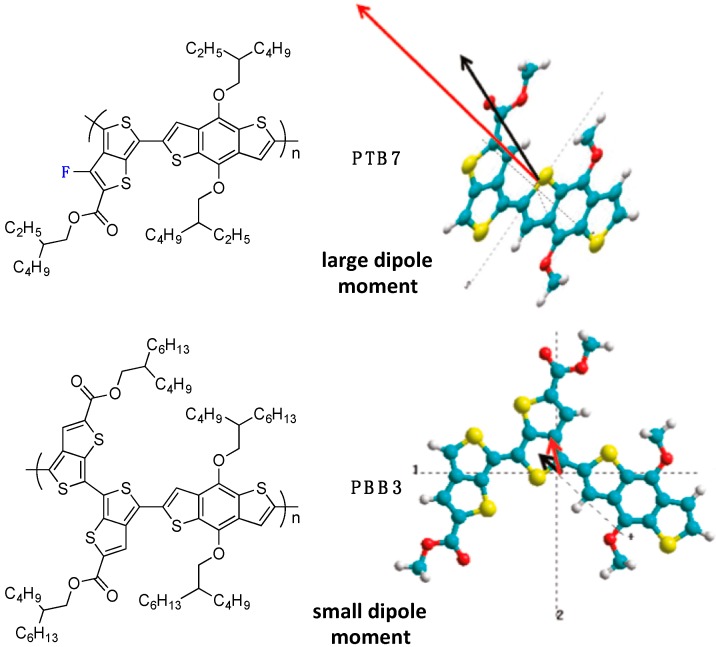
Chemical structure of the PTB7 and PBB3 copolymer (**left**). Dipole moment calculations for PBB3 and PTB7 (**right**). The ground (black arrow) and excited (red arrow) state dipole moments of the PBB and PTB monomer units are drawn to scale. The axes (dotted lines) represent the center of mass axes in the x, y and z directions. The vector of the dipole shows the direction of electron flow in these directions (adapted with permission from [57]; Copyright 2011 American Chemical Society).

This paragraph presents some experimental evidence of the impact of the backbone fluorination on charge carrier generation and recombination. One of the first reports on the influence of fluorination on charge generation is from Yu *et al.* in 2011 [57]. In their contribution, the authors mainly focus on the different behavior between the highly performing fluorinated **PTB7** copolymer (PCE of 7.4%) with the far lesser performing non-fluorinated **PBB3** copolymer (PCE of 2.04%) (Figure 15) despite otherwise promising properties (optimal band-gap, frontier energy level positioning and blend morphology).

We note that **PTB7** and **PBB3** have similar donor and acceptor building blocks (except for the fluorine atom on the thieno[3,4-*b*]thiophene unit) but differing conjugated backbones as well as different solubilizing side-chains (for **PTB7**, R_1_ = R_2_ = 2-ethylhexyl, while for **PBB3**, R_1_ = R_2_ = 2-butyloctyl). Nevertheless, based on complementary transient absorption spectroscopy, **PTB7** was found to exhibit a lower exciton lifetime than **PBB3**, while its charge transfer state lifetime was larger. The authors suggest that the large change in the dipole moment localized on the single fluorinated thienothiophene unit in **PTB7** renders the excited state highly polarized when compared to that of **PBB3**. Consequently, the negative charge tends to be more localized in this unit, facilitating electron transfer to the PCBM acceptor, while the positive charge remains localized on the electron-rich benzodithiophene unit. In contrast, the lower dipole moment of **PBB3** favors recombination.

**Figure 16:**
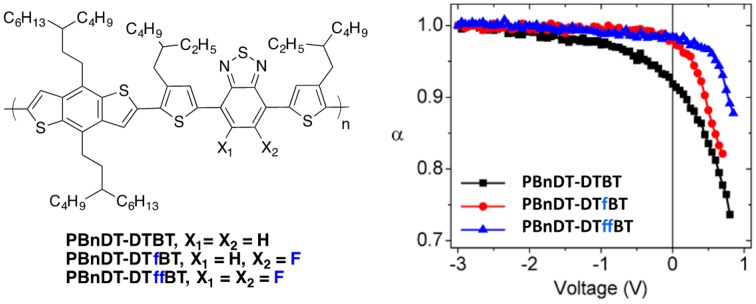
Chemical structures of the investigated polymers (**left**) and variation of α as a function of the voltage in solar cells with different incident power light (**right**) (see text for details) (adapted with permission from [59]; Copyright 2013 American Chemical Society).

A similar analysis has been made by You and co-workers on a series of **PBnDT-DTBT**-based polymers (Figure 16) [59].

The change in the calculated dipole moment from ground state to excited state when comparing **PBnDT-DTB** to **PBnDT-DTffBT** is shown to increase with fluorination and is tentatively associated with a decrease in the exciton binding energy and is considered as a possible driving force for enhanced charge separation. In the same study, the photocurrent (*J*_photo_) was measured as a function of the incident light intensity (*P*_light_) and the power-law scaling exponent α, defined by:

(1) $$J_{\text{photo}}=\beta {\left(P_{\text{light}}\right)}^{\alpha }$$

which could be estimated as a function of the applied voltage (Figure 16).

If the bimolecular recombination losses are negligible, the number of charge carriers collected (*i.e.*, the photocurrent) should scale linearly with the light intensity, *i.e.*, α should be equal to one [84,85]. As can be seen in Figure 16, α deviates substantially from one at maximum power (around 0.5 V), revealing significant carrier recombination. This deviation is minimized for fluorinated polymers. Moreover, while at short-circuit current conditions both fluorinated polymers exhibit an α value close to one (negligible bimolecular recombination losses at higher electric fields), the non-fluorinated polymer needs a bias value of −3 V (*i.e.*, a stronger internal electric field) for α to reach one. The authors therefore could conclude that the losses due to bimolecular recombination are sensitive to the fluorine substituents on the polymer backbone.

**Figure 17:**
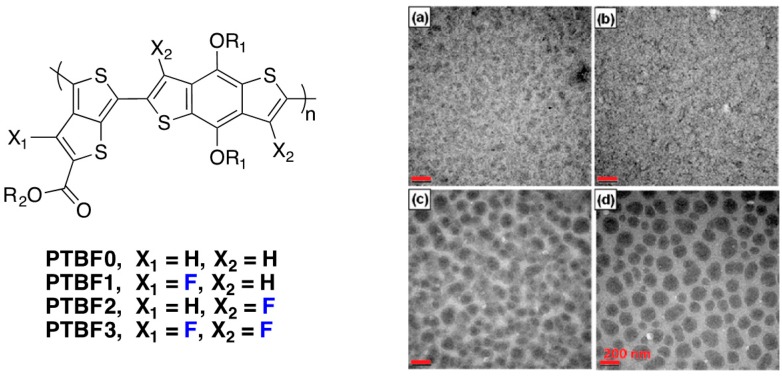
Chemical structures of the investigated polymers (**left**) and TEM images (**right**) of polymer/PC_71_BM films prepared from DCB solvent: PTBF0 (**a**); PTBF1 (**b**); PTBF2 (**c**); and PTBF3 (**d**). Scale bar = 200 nm (adapted with permission from [25]; Copyright 2011 American Chemical Society).

Another study on the influence of fluorination on charge generation and extraction has been published by Yu and co-workers [25]. They synthesized four different polymers with different degrees of fluorination and observed, by Transmission Electron Microscopy (TEM), different morphologies when blended with PC_71_BM from DCB solution (Figure 17).

Even though the side chains are different for the different polymers (R_1_ = n-octyl for **PTBF0** and 2-ethylexyl for the other polymers), it appears clearly that macrophase separation can be observed in blends using **PTBF2** and **PTBF3** with no percolation path for the PCBM rich domains (dark regions in Figure 17). Due to the limited exciton diffusion length (around 10 nm), photogenerated excitons are expected to recombine very efficiently before reaching a donor/acceptor interface in the case of **PTBF2** and **PTBF3**. This is in-line with the decrease in photocurrent observed in solar cells elaborated from these two polymers. Moreover, free charge carriers will also be difficult to extract in a macrophase separated blend. The absence of percolation pathway for the electrons will induce a strong electric-field dependence of the photocurrent resulting in a low *FF* [86] as also observed by the authors for **PTBF2** and **PTBF3** [25]. Finally, the bimolecular recombination expected to be efficient in macrophase separated blends will decrease the *V*_oc_ [87] as mentioned by the authors for **PTBF2** and **PTBF3** [25]. The morphology in blends is well correlated with the planarity of the polymer backbone upon fluorination as measured by X-Ray diffraction [25]. The improved planarity of the backbone of the more fluorinated polymers favours the development of long-range order as well as the exclusion of the fullerene [88]. Moreover, the fluorinated backbones introduce fluorophobicity for PC_71_BM molecules, favouring again the phase separation [25].

**Figure 18:**
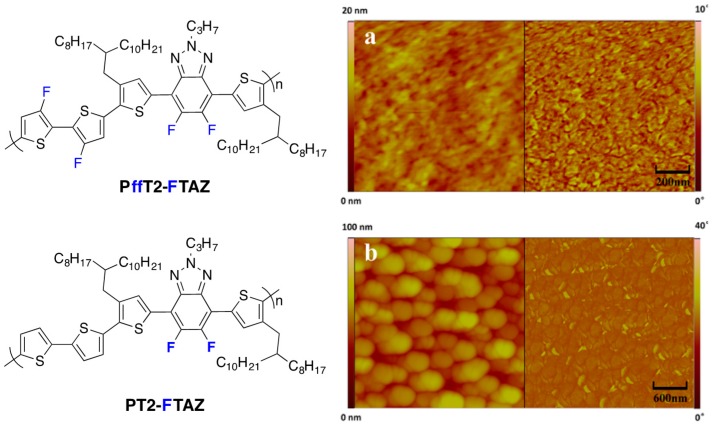
Chemical structures of the investigated polymers (**left**) and AFM image of PffT2-FTAZ/PC_71_BM blend (**a**) and PT2-FTAZ/PC_71_BM blend (**b**). The AFM surface topography and phase images are displayed on the left and on the right, respectively (Adapted with permission from [32]. Copyright 2015 Elsevier).

This result is in apparent contradiction with a recent study of Yan *et al.* discussing blend morphologies as a function of fluorine density along the conjugated polymer backbone (Figure 18) [32].

Even though this study does not deal specifically with charge generation and recombination, the overall PCE was much higher using **PffT2-FTAZ** polymer (7.8% compared to 2.8% for the di-fluorinated polymer). Interestingly, only the less-fluorinated backbone led to a macrophase separation (Figure 18). This result is in apparent contradiction with the less-well-performing highly fluorinated polymer **PTBF3** presented above [25] and is tentatively explained by the authors with the fluorine density in the repetition unit and with the absence of fused aromatic units in **PffT2-FTAZ**. Indeed, **PTBF3** has a perfluorinated backbone while **PffT2-FTAZ** has four fluorine and four hydrogen atoms in each repeating unit and, therefore, the fluorophobicity might be less pronounced in **PffT2-FTAZ**. Moreover, the fused aromatic units in **PTBF3** combined with the planarization induced by fluorine can favor long-range ordered structure with large polymer domain size.

**Figure 19:**
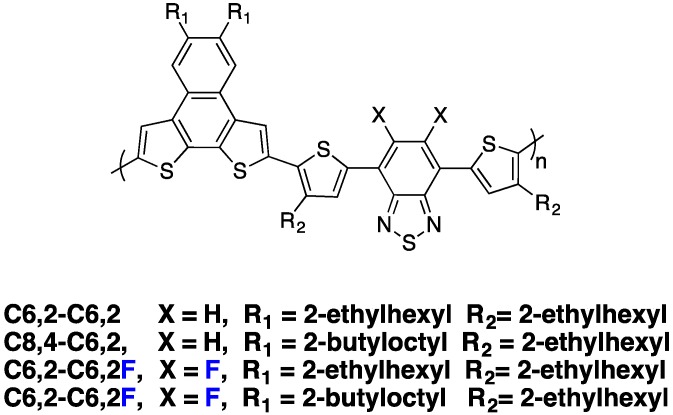
Chemical structures of the investigated polymers [71].

In a recent publication, You and co-workers intended to separate the influence of the solubilizing alkyl side-chains from the impact of the fluorination of the backbone (Figure 19) [71].

The influence of the fluorination of the polymer backbone on the morphology of the blend is qualitatively comparable to the one mentioned above, with an increase in domain size for fluorinated polymers (AFM and X-Ray scattering experiments). The authors note that in this series of polymers, fluorine introduction into the conjugated backbone increases the charge collection probability at all voltages leading to an observed increase in *FF* upon fluorination. As in-depth X-Ray scattering experiments and charge carrier mobility measurements are not in line with this observation, the authors claim that the higher domain purity evidenced for fluorinated polymers in blends could be responsible for the better charge collection using these polymers [89]. They also mention that the introduction of the most electronegative fluorine element could create a strong internal dipole moment that will lower the Coulombic potential between e-h pairs [57].

**Figure 20:**
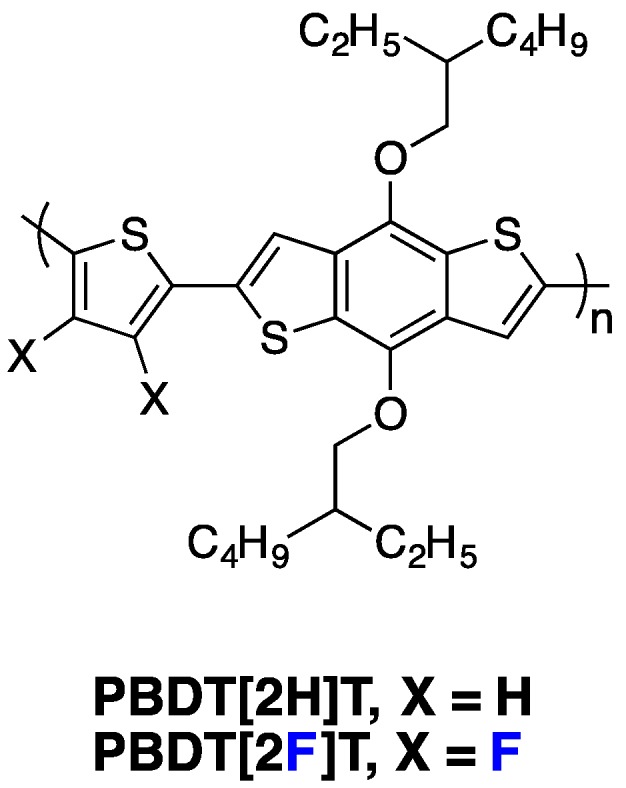
Chemical structures of the investigated PBDT[2X]T polymers [31].

Another series of polymers was recently introduced by Beaujuge and co-workers (Figure 20) [31].

For these materials, no difference in blend morphology was observed either by transmission electron microscopy or by photoluminescence quenching between the fluorinated and the non-fluorinated polymer. Indirect electric measurements (photocurrent measurements as a function of the effective voltage) suggest, nevertheless, that charges can be more efficiently separated and extracted in **PBDT[2F]T**.

Using benzotriazole-based monomer coupled on a standard benzodithiophene, You and co-workers have mentioned an interesting relationship between bimolecular recombination and *d*-spacing measured by XRD on polymer films [90]. They observed that the fluorine atoms create a repulsion strength towards hydrocarbon materials as observed in their fluorinated polymers series, in which the *d*-spacing value increases with the fluorine content. They speculate that similar behavior should be observed between fluorinated polymers and PCBM derivatives keeping away the PCBM molecules from the polymer chains. This would increase the electron-hole charge transfer complex separation and thus slow down bimolecular recombination.

This last example brings us to conclude that the field of charge generation and charge extraction in fluorinated copolymers is a quite open topic, as these phenomena depend strongly on the not yet well understood blend morphology.

## 7. *n*-Type Fluorinated Polymers

**Figure 21:**
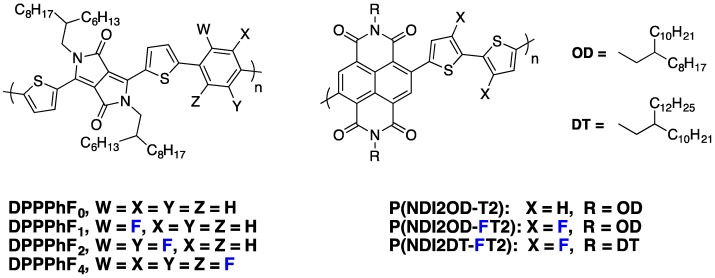
Chemical structures of *n*-type fluorinated copolymers.

The design of new *n*-type conjugated materials, with especially improved photon harvesting abilities to replace PCBM-fullerene derivatives in PSCs, is another issue of significant importance. If small molecules appear highly promising in particular through their 3D-edifice building ability [91,92], the best photovoltaic performances are still achieved with *n*-type copolymers. In particular, polymers using the strong electron withdrawing naphthalene di-imide (NDI) unit in the conjugated backbone have shown high electron mobilities and strong absorption between 500 and 750 nm, leading to high PCEs in all polymer solar cells [15,93]. As discussed previously in this review, due to its strong electronegativity, the addition of fluorine atoms along the conjugated backbone allows the lowering of both FMO energy levels. Moreover, it also provides planarization of the conjugated backbone through heteroatom interactions, leading to a higher degree of charge delocalization. These two features could be of interest to design new organic *n*-type semiconducting materials. Along these lines, Jo and Thelakkat groups have shown that Diketopyrrolopyrrole-based polymers using fluorine-flanked π-linkers between two adjacent DPP moieties lead to higher electron mobilities in comparison to non-fluorinated ones [29,94]. Interestingly, Jo and co-workers have shown, on the **DPPPhF_n_** copolymer series (Figure 21), that increasing the number of fluorine substitutions does not only impact the LUMO level, which decreases slightly, but it also modifies the polymer chain orientation in thin films, with a face-on orientation becoming more favorable. A high electron field-effect mobility of around 2.3 cm^2^·V^−1^·s^−1^ has been measured for **DPPPhF_4_**.

Recently, Jo and co-workers applied the fluorination strategy to one of the most investigated *n*-type copolymers, the **P(NDI2OD-T2)** derivative (Figure 21) [95]. Amazingly, in their specific case, the authors observed, by cyclic voltammetry, a raising LUMO level upon backbone fluorination of about 0.15 eV (Table 6). The authors mentioned an expected beneficial impact on the *V*_oc_ but did not give an explanation for this particular behavior. In blends with an electron-donor fluorinated PTB derivative (**PBDTT-TT-F**), the fluorinated derivative led to significant PCE increase in inverted solar cell devices (Table 6). Although the *V*_oc_ slightly increased, the main improvements were measured on *J*_sc_ and *FF*. The authors explained the increase of these parameters by the superior exciton quenching efficiency and charge carrier transport capability, as well as by the reduced bimolecular recombination. In addition, they also observed by GIWAXS characterizations that the fluorinated **P(NDI2OD-FT2)** derivative forms highly crystalline nanostructures with a preferential face-on orientation, which should favor the charge transport across the bulk of the active layer. A further chemical engineering on NDI side-chains has allowed the authors to reach the high PCE of 6.7% with the **P(NDI2DT-FT2)** derivative.

**Table 6:** The *n*-type polymer properties and photovoltaic device parameters.

Polymer	LUMO (eV)	Eg_Opt_ (eV) ^a^	*V*_oc_ (V)	*J*_sc_ (mA·cm^−2^)	*FF* (%)	PCE (%)	µ_e_ (cm^2^·V^−1^·s^−1^)
P(NDI2OD-T2)	−4.05	1.44	0.79	12.3	56	5.28	1.2 × 10^−5^
P(NDI2OD-FT2)	−3.91	1.59	0.81	11.9	63	6.29	3.9 × 10^−4^
P(NDI2DT-FT2)	-	-	0.81	13.5	62	6.71	5.5 × 10^−4^

^a^ From UV–visible absorption onset in solid state.

These series of pioneering fluorinated *n*-type polymers demonstrate that similar positive impacts could be expected from backbone fluorination for both components of the PSC active layer and thus pave the way towards all fluorinated polymer solar cells.

## 8. Conclusions

In this review, we presented the influence of backbone fluorination of π-conjugated polymers for photovoltaic applications. The trend observed systematically for such polymers is a lowering of their HOMO when compared to their non-fluorinated analogues. The lowering of the HOMO is of particular interest in photovoltaic applications, as the open circuit voltage (*V*_oc_) increases when the HOMO of the electron-donor polymer is lowered. The introduction of the very electronegative fluorine atom into the polymer backbone also almost systematically lowers the LUMO of the polymer. If the LUMO is lowered as much as the HOMO (as is often observed), the energy band-gap and therefore the absorption wavelength range of the fluorinated polymer will remain comparable to that of its non-fluorinated analogue. An interesting auxochromic effect of the fluorine atom has sometimes been observed with a significant extinction coefficient improvement for fluorinated polymers.

On the other hand, tuning the frontier energy levels is a necessary but insufficient condition to reach high power conversion efficiency. The second generally observed trend when fluorine is introduced on the polymer backbone is the planarization of the backbone. The origin for this planarization is still under discussion, but the consequences have been clearly identified: fluorinated polymers tend to aggregate (even in solution) and the morphology they adopt in thin films when blended with PCBM is different from that obtained with their non-fluorinated analogues. We tentatively presented in this review the influence of fluorination on several macro-properties that are closely linked to each other: the morphology in thin films (pure polymer and blends), the charge carrier mobility (pure polymer and blends) and the charge carrier generation and recombination kinetics (blends). At this stage, it seems impossible to draw a complete picture of the influence of fluorination on each of these properties as some contradictory reports can be found in the literature. However, it is established that fluorinated electron-donor polymers presenting highly interesting properties for photovoltaic applications have been synthesized. The tendency of fluorinated polymers to aggregate as well as the fluorophobicity of PC_71_BM molecules can be overwhelmed to avoid macrophase separation in thin films. Moreover, domains with a high degree of purity and an adequate microstructure can be obtained with fluorinated polymers. It has also been proven that fluorinated electron-donor polymers can lead to very high hole mobility, and well-balanced and reasonably high charge carrier mobility in blends with PCBM has been measured in the substrate plane as well as in the direction perpendicular to the substrate. Experimentally, high free charge carrier generation rates as well as limited bimolecular recombination have been measured in blends based on fluorinated materials as electron-donor polymers. Photovoltaic cells elaborated with an electron-donor polymer presenting all the positive properties mentioned above will lead to high short-circuit current density (*J*_sc_), open-circuit voltage (*V*_oc_) and fill-factor (*FF*) values. Fluorinated electron-donor polymers are very promising candidates for this ideal polymer, as impressive photovoltaic parameters (*J*_sc_ = 18.8 mA/cm^2^; *FF* = 75%; *V*_oc_ = 0.77 V) with an overall PCE of 10.8% have been recently reported for such a polymer [4].

Nevertheless, as exposed in the present review, complementary experimental and theoretical investigations are still needed in the field of fluorinated polymers in order to fully understand the effect of fluorine introduction into the backbone on interlinked optoelectronic and structural properties. By continuing these efforts, further increase in the photovoltaic device performances may be expected.
